# Appraisal of the diagnostic procedures of acute pancreatitis in the guidelines

**DOI:** 10.1186/s13643-020-01559-4

**Published:** 2021-01-09

**Authors:** Ke-Qian Yi, Ting Yang, Yan-Min Yang, Guo-Li Lan, Li-Ya An, Yu-Xing Qi, Hong-Bo Fan, Yong-Qing Duan, Da-Li Sun

**Affiliations:** 1 Department of Gastrointestinal Surgery, Second Affiliated Hospital of Kunming Medical University/Second Faculty of Clinical Medicine, Kunming Medical University, Kunming, 650101 China; 2 Digestive System Department, People’s Hospital of Yuxi, Yuxi, 653100 China

**Keywords:** Severe pancreatitis, Guidelines, Diagnosis

## Abstract

**Objectives:**

The purpose of this study was to comprehensively assess the heterogeneity of procedures in the diagnostic guidelines for acute pancreatitis and to identify gaps limiting knowledge in diagnosing this disease.

**Methods:**

A systematic search of a number of databases was performed to determine the guidelines for the diagnosis of acute pancreatitis in patients with severe pancreatitis. The guidelines for the diagnosis of severe pancreatitis were evaluated by AGREE II. The Measurement Scale of Rate of Agreement (MSRA) was used to assess the guidelines (2015–2020) and extract evidence supporting these recommendations for analysis.

**Results:**

Seven diagnostic guidelines for acute pancreatitis were included. Only the 2019 WSES Guidelines for the Management of Severe Acute Pancreatitis and the Japanese Guidelines for the Management of Acute Pancreatitis: Japanese Guidelines 2015 had a total score of more than 60%, which is worthy of clinical recommendation. The average scores of the Scope and Purpose domain and the Clarity and Expression domain were the highest at 71.62% and 75.59%, respectively, while the average score of the Applicability area was the lowest at 16.67%. The included guidelines were further analyzed to determine the heterogeneity of the diagnosis of acute pancreatitis. The main reasons for the heterogeneity were the citation of low-quality evidence, the presence of far too many indicators for the classification of acute pancreatitis, unclear depictions of the standard, and poorly comprehensive recommendations for the diagnosis of the aetiology in the primary diagnosis of acute pancreatitis, the severity classification, the aetiological diagnosis, and the diagnosis of comorbidities.

**Conclusions:**

The quality of different diagnostic guidelines for severe pancreatitis is uneven. The recommendations are largely based on low-quality evidence, and the guidelines still have much room for improvement to reach a high level of quality. The diagnostic procedures for acute pancreatitis vary widely in different guidelines. There are large differences between them, and resolving the abovementioned reasons would be a very wise choice for guideline developers to revise and upgrade the guidelines in the future.

**Supplementary Information:**

The online version contains supplementary material available at 10.1186/s13643-020-01559-4.

## Background

Acute pancreatitis is an inflammatory process that can lead to local and systemic inflammatory reactions. Globally, the incidence of acute pancreatitis is increasing and is expected to further increase medical burden [1]. Although most cases of acute pancreatitis are mild, approximately 20–30% of patients will develop severe pancreatitis with pancreatic tissue necrosis and/or multiple organ failure. In a systematic review and meta-analysis of 6970 patients, the mortality rate of patients with infectious necrosis and organ failure was 35.2%, while the mortality rate of patients with concurrent aseptic necrosis and organ failure was 19.8%. If the patient had infectious necrosis but no organ failure, the mortality rate was 1.4% [2].

A standardized diagnosis of acute pancreatitis is essential for the early treatment of the disease. In recent years, many academic organizations and experts in related fields have developed guidelines for the diagnosis of acute pancreatitis [2–8]. The diagnosis of inflammation should follow proper diagnostic procedures to ensure that the diagnosis of acute pancreatitis is logical and reasonable [3]. However, the current diagnostic guidelines for acute pancreatitis vary widely in terms of the recommendations and corresponding evidence supporting acute pancreatitis, which limits their clinical use.

Therefore, this study intends to adopt the The Appraisal of Guidelines for Research and Evaluation II (AGREE II) tool to comprehensively evaluate the current guidelines for the diagnosis of acute pancreatitis, comb the diagnostic procedures for acute pancreatitis, and analyse the differences among the recommendations and supporting evidence for the diagnostic steps for acute pancreatitis in the current guidelines so that future guideline developers can make meaningful improvements and updates.

## Methods

### Study design

This study was developed through international collaboration and discussion with a panel of experts composed of managers engaged in emergency surgery and patients with severe acute pancreatitis.

### Data sources and literature selection process

Diagnostic studies were searched in the PubMed, Ovid, Web of Science, Science Direct, CNKI, and Wanfang databases (2015–2020). Keywords include the following: “Severe Pancreatitis,” “Pancreatitis,” “SAP,” “Acute Pancreatitis”; “Guidelines,” “Recommendations,” “Consensus.” “Statements.” The terms were first retrieved individually and jointly. Then, the set operator “and” was used to combine the results with studies identified through the term “diagnosis.” References that were included in the guidelines were manually retrieved to identify potential additional guidelines.

### Selection of guidelines

Inclusion criteria were as follows: (1) guidelines for the diagnosis of severe pancreatitis; (2) a complete guideline text, published in English or Chinese; and (3) the latest version of a set of guidelines that has been updated multiple times.

The exclusion criteria were as follows: (1) reviews and comments on the diagnosis of severe pancreatitis; (2) interpretations of the guidelines for severe pancreatitis; (3) repeated publication guidelines; (4) non-English- and non-Chinese language guidelines; and (5) guidelines whose full contents could not be retrieved.

### Quality evaluation of guidelines

We used the latest version of the AGREE II tool to evaluate each set of acute pancreatitis guidelines that met our inclusion criteria [9]. According to the AGREE II manual, each set of guidelines is scored on 23 items in 6 domains. Domain 1 (Scope and Purpose) is divided into three items: the guidelines provide a clear description of their general purpose, the health issues covered by the guidelines are clearly described, and the applicable population of the guideline (patients, public, etc.) are clearly described. Domain 2 (Stakeholder Participation) is based on three items: the guideline development group is composed of individuals from relevant fields, the target population has been asked about the guidelines, and the target users are clearly described. Domain 3 (Rigorousness of Development) includes 8 items: the guidelines provide a systematic method for retrieving evidence, the evidence selection criteria are clearly described, the strength and limitations of evidence are clearly described, the method of forming a recommendation is clearly described, recommendations are formulated considering the health benefits and side effects, clear links are made between recommendations and supporting evidence, the guidelines have been externally review by experts prior to publication, and steps are provided to develop guidelines updates. Domain 4 (Clarity and Expression) includes three items: recommendations are clear and unambiguous, different options or health issues are clearly listed, and major recommendations are easily identified. Domain 5 (Applicability) includes 4 items: the guidelines describe the facilitators and obstacles in the application, the guidelines provide advice and/or tools for applying recommendations, the guidelines take into account relevant resources that may be needed in the application of recommendations, and the guidelines provide standards for monitoring and/or auditing. Domain 6 (Editor Independence) is based on two items: the sponsor’s point of view does not affect the content of the guideline, and the conflicts of interests of members of the guideline development organization are documented and publicized.

In this study, four reviewers (K.Q.Y., T.Y., Y.M.Y., and H.B.F.) scored each guideline for the diagnosis of severe pancreatitis based on the AGREE II user manual, independently evaluated the search results to determine whether to include or exclude references, and extracted the general characteristics of each guideline. If there were differences, they were resolved through consultation and mediation or consultation with other expert reviewers. Among these four reviewers, Y.M.Y., H.B.F., Y.Q.D., and D.L.S. are responsible for the clinical diagnosis and treatment of severe pancreatitis. In addition, D.L.S. and T.Y. have extensive experience in applying AGREE II. All authors have undergone standardized training on the AGREE II tools.

The AGREE II user manual defines each item and assists the user in scoring the guidelines. The items are scored on a scale ranging from 1 (no item) to 7 (high-quality items). The scores are summed and then normalized to the percentage of the maximum possible score, and the domain score is calculated. The overall assessment includes whether the guideline is recommended for clinical practice. In general, the AGREE II group divides the overall assessment into three categories: recommended, revised recommended, and not recommended. Consensus was reached based on the results of 23 evaluations and the reviewers’ overall judgment. A guide was classified as “Recommended” if it achieved a total score of > 60%, “Revised recommended” if it achieved a total score of 30–60%, and “Not recommended” if it achieved a total score of < 30%.

### Evaluation of recommendations and evidence for severe pancreatitis

To analyse the heterogeneity of the recommendations and the evidence for the diagnosis of severe pancreatitis among the guidelines, we used the Oxford Centre for Evidence-based Medicine-Levels of Evidence (OCEBM) evidence grading system (Table S1) to reclassify the recommendations and corresponding evidence in the original guidelines. The recommendations for severe pancreatitis in the guidelines with higher scores and the more comprehensive recommendations by the AGREE II tool were used to establish a recommendation distribution table for each guideline, and the similarity of the same item was compared if it was recommended in at least 4 guidelines. The highest level of evidence supporting this recommendation was also extracted and analyzed. In each module, the consistency rate among these guidelines was scored using the Measurement Scale of Rate of Agreement (MSRA) [10]. The evidence was reclassified using the OCEBM grading system. Details of the evidence classification method are shown in Table S1.

### Data analysis

We used a descriptive statistical analysis method to calculate the standard score for each domain, expressed as a percentage, and also list the median and range for each domain score. In addition, the distribution of the number of recommendations and the level of evidence is also expressed as a percentage. Two-way ANOVA was used to test the consistency between each reviewer’s scores using the within-group correlation effects of single-factor raters. According to previous studies, a degree of consistency between 0.01 and 0.20 is considered slight, 0.21–0.40 is considered moderate, 0.41–0.60 is considered moderate, 0.6–0.80 is considered substantial, and 0.81–1.00 is considered very good [11]. *P* < 0.05 was considered statistically significant. SPSS version 17.0 (SPSS, Chicago, USA) was used to conduct statistical analysis.

## Results

### Guide features

**Fig. 1:**
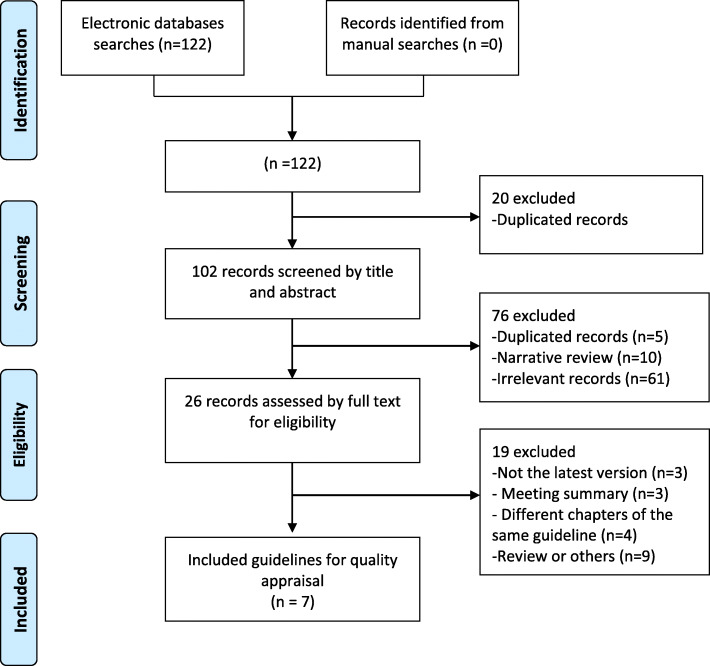
Flow chart of study selection

A total of 122 related studies were retrieved, and some guidelines or consensuses were excluded for a variety of reasons. Finally, only seven guidelines or consensuses were included (Fig. 1). The basic characteristics of the included guidelines are shown in Table 1. The publication date of the included guidelines ranged from 2014 to 2020. Two of the articles [2, 6] were from Italy, three [3, 7, 8] were from China, and the remaining two were from Japan and Canada [4, 5]. One article [2] presented the original version of the guidelines, and the remaining six [3–8] presented updated versions that improved and extended the guidelines. Of the seven included guides, five guides used three grading systems to rate the level of evidence and the strength of the recommendation (Table 1); 3 [2, 5, 8] adopted the Grading of Recommendations Assessment Development and Evaluation (GRADE) system, one [6] used the APACHE system, and one used the Delphi classification standard [7]. The evidence levels and recommended strength codes in the different rating systems vary widely.

**Table 1:** Description of the guidelines included in this study

Guideline ID	Short name	Year	Country	Reference number	Funding	Grading system	Interest conflict	Version
Ari Leppäniemi et al. [2]	Ar	2019	Italy	142	Not applicable	GRADE	No	First
Li JX et al. [3]	Li	2017	China	50	NG	NG	No	Updated
Joshua A. Greenberg, et al. [4]	Jo	2015	Canada	137	NG	NG	None declared	Updated
Masamichi Yokoe et al. [5]	Ma	2015	Japan	339	Ajinomoto	GRADE	No	Updated
AISP [6]	AI	2015	Italy	219	NG	APACHE	None declared.	Updated
GSCCSITWM [7]	GS	2014	China	18	NSTSP	Delphi	NG	Updated
PDGCSG et al. [8]	PD	2019	China	61	NG	GRADE	NG	Updated

*AISP* The Italian Association for the Study of the Pancreas; *GSCCSITWM* General Surgery Committee of Chinese Society of Integrated Traditional and Western Medicine; *PDGCSG* Pancreatic Disease Group of Chinese Society of Gastroenterology; *Ajinomoto* Ajinomoto Pharmaceuticals Co., Ltd., Tokyo, Japan; *NSTSP* National Science and Technology Support Program; *NO* there is no interest conflict; *NG* not given

### Quality evaluation of acute pancreatitis guidelines

The guidelines were reviewed based on the latest English version of the AGREE II instrument (2017 edition) [9], a validated assessment tool designed to provide a framework for evaluating and monitoring clinical guidelines that can be used to measure and quantify guideline quality. The results of evaluating the methodological quality of the included guidelines using the AGREE II tool are shown below. Table 2 indicates that the Scope and Purpose domain and the Clarity and Expression domain reached a relatively significant median score of 71.62% (range 44.44–87.50%) and 75.59% (range 23.61–94.44%), respectively. The average scores of the Stakeholder Participation, Rigorousness of Development, and Editor Independence domains were roughly similar, 56.15% (range 33.33–77.78%), 58.70% (range 43.22–79.17%), and 54.17% (range 0.00–97.92%), respectively. Unfortunately, the average score of the Applicability domain was the lowest at 16.67% (range 0.00–34.38%). Finally, according to the scores, we provided overall recommendations. The detailed overall scores for each guideline are listed in Table 2. There were three guides with overall evaluation scores between 30 and 60% [4, 6, 8]. These guidelines fell into the recommended category, but they still need to be improved. Two guidelines had a total score of less than 30% [3, 8], and so they could not be recommended. It is worth noting that there were two guidelines with relatively high scores (exceeding 60%) in each area except the Applicability domain [2, 5],which were classified according to the recommendations of clinical practice developed by the World Society of Emergency Surgery (WSES) and the Japanese Society of Hepato-Biliary-Pancreatic Surgery, respectively. In this study, intra-class correlation coefficient (ICC) values were used to evaluate the consistency of the four reviewers’ assessments of the diagnosis guidelines for acute pancreatitis and were all found to exceed 0.90 (Table 2), indicating that the appraisers’ evaluations were remarkably consistent.

**Table 2:** AGREE II domain score and ICC of the included guidelines

Guideline	Scope and Purpose	Stakeholder Involvement	Rigour of Development	Clarity and Expression	Applicability	Editorial Independence	Overall Assessment	ICC(mean±SD)
Ar [2]	87.5%	66.7%	79.2%	94.4%	34.4%	95.8%	65.9%	0.998
Li [3]	44.4%	58.3%	46.4%	23.6%	3.1%	50.0%	28.2%	0.996
Jo [4]	84.7%	38.9%	49.5%	55.5%	16.7%	41.7%	35.9%	0.995
Ma [5]	84.7%	77.8%	64.1%	87.5%	50.0%	97.9%	72.0%	0.965
AI [6]	50.0%	58.3%	66.7%	88.9%	0.0%	45.8%	38.7%	0.997
GS [7]	63.8%	33.3%	43.2%	84.7%	12.5%	0.00%	29.7%	0.997
PD [8]	86.1%	59.7%	62.0%	94.4%	25.0%	47.9%	57.8%	0.989
Median score	71.6%	56.2%	58.7%	75.6%	16.7%	54.2%	46.9%	–
(range)	(44.4–87.5%)	(33.3–77.8%)	(43.2–79.1%)	(23.6–94.4%)	(0.00–34.4%)	(0.00–97.9%)	(28.2–72.0%)	–

### Heterogeneity of recommendations and evidence for the diagnosis of acute pancreatitis (Tables 3 and 4 and Fig. 2)

#### Primary diagnosis of AP

**Table 3:** Recommendations for the diagnosis of acute pancreatitis in the included guidelines (2014–2020)

Guideline	Reference Standard	Ar [2]	Li [3]	Jo [4]	Ma [5]	AI [6]	GS [7]	PD [8]
(1) Primary diagnosis of AP								
Symptoms and signs	Abdominal pain (acute onset of a persistent, severe, epigastric pain often radiating to the back)	●	●	–	–	–	●	●
Biochemical evidence	Serum lipase activity (or amylase) at least 3 times greater than the upper limit of normal	●	●	●	●	–	●	●
	Urinary trypsinogen-2 dipstick				О			
Characteristic findings from abdominal imaging	Characteristic findings of acute pancreatitis on ultrasound (US), computed tomography (CT), or magnetic resonance imaging (MRI)	●	●	●	●	–	●	●
(2) Classification of the severity of AP								
Classification tools	Revised Atlanta Classification	●	●	●	–	–	●	●
	Determinant-based Classification	●	–	–	–	–	–	–
	JPN Severity Score	–	–	–	●	–	●	–
Prognostic factors	C-reactive protein (CRP) level ≥ 150 mg/l	●	●	●	–	–	–	●
	Urea > 20 mg/dl	●	–	–	–	–	●	●
	CT Severity Index	●	●	–	–	–	–	–
	Bedside index of severity of acute pancreatitis (BISAP) score	●	–	–	●	–	–	●
	Acute Physiology and Chronic Health Evaluation II (APACHE-II) score	●	●	●	–	–	●	●
(3) Etiological diagnosis of AP								
	Ultrasound (US) / endoscopic ultrasound (EUS)	●	●	●	●	●	●	●
	Contrast-enhanced computed tomography (CECT)	●	–	–	●	–	–	–
	Magnetic resonance imaging (MRI)/magnetic resonance cholangiopancreatography (MRCP)	●	●	●	●	●	–	●
	Endoscopic retrograde cholangiopancreatography (ERCP)	●	●	●	●	–	–	●
	Serum triglyceride levels ≥11.3mmol/L	●	–	–	–	–	●	●
	Genetic testing	–	–	–	–	–	●	–
(4) Diagnosis of comorbidities of AP								
	CECT	●	●	●	●	●	●	●
	contrast-enhanced MRI	–	–	–	–	●	●	–
	US	–	●	–	–	–	–	–
Pancreatic necrosis/abdominal fluid collection/pseudocyst / walled-off necrosis (WoN)	Fine needle aspiration (FNA)	●	●	●	●	●	●	●
Infected collection, necrosis or WoN	MRCP	–	–	–	–	●	–	–
pancreatic fistula	sustained intra-abdominal pressure (IAP) > 20 mmHg	–	●	–	●	●	–	●
Abdominal compartment syndrome (ACS)	CECT	–	–	●	●	●	–	–
Vascular complications	Hematocrit > 44%	●	–	–	–	–	●	●
Risk factors of pancreatic necrosis	Procalcitonin	●	●	–	–	–	–	●
	Calcium levels	●	–	–	–	–	–	●

О urinary trypsinogen-2 dipstick may be useful for minimally invasive method and rapid diagnosis of acute pancreatitis. However, this is not commercially available in Japan and therefore it cannot be recommended in the guideline

Black circle indicates being recommended definitely; gray circle indicates being mentioned; en dash indicates being not mentioned

*APACHE*-*II* Acute Physiology and Chronic Health Evaluation II score

**Table 4:** Scientific agreement of formulated recommendations for acute pancreatitis diagnosis in the included guidelines (2014–2020)

Guideline	Reference standard	Ar [2]	Li [3]	Jo [4]	Ma [5]	AI [6]	GS [7]	PD [8]
(1) Primary diagnosis of AP								
Symptoms and signs	Abdominal pain (acute onset of a persistent, severe, epigastric pain often radiating to the back)	–	80–100%	–	–	–	80–100%	80–100%
Biochemical evidence	Serum lipase activity (or amylase) at least 3 times greater than the upper limit of normal	–	80–100%	80–100%	80–100%	–	80–100%	80–100%
Characteristic findings from abdominal imaging	Characteristic findings of acute pancreatitis on ultrasound, computed tomography or magnetic resonance imaging	–	80–100%	80–100%	80–100%	–	80–100%	80–100%
(2) Classification of the severity of AP								
Classification tools	Revised Atlanta Classification	–	80–100%	80–100%	–	–	80–100%	80–100%
	C-reactive Protein(CRP) level ≥ 150 mg/l	–	40–60%	80–100%	–	–	–	80–100%
	APACHE-II score	–	80–100%	80–100%	–	–	80–100%	80–100%
(3) Etiological diagnosis of AP								
	Ultrasound (US) / endoscopic ultrasound (EUS)	–	80–100%	80–100%	80–100%	20–40%	40–60%	80–100%
	Magnetic resonance imaging (MRI) / Magnetic resonance cholangiopancreatography (MRCP)	–	80–100%	80–100%	80–100%	80–100%	–	80–100%
	Endoscopic retrograde cholangiopancreatography (ERCP)	–	80–100%	80–100%	80–100%	–	–	80–100%
(4) Diagnosis of comorbidities of AP								
pancreatic necrosis/ abdominal fluid collection/pseudocyst / walled-off necrosis (WoN)	CE-CT	–	80–100%	40–60%	80–100%	80–100%	80–100%	80–100%
Infected collection, necrosis or WoN	Fine needle aspiration (FNA)	–	80–100%	80–100%	80–100%	80–100%	80–100%	20–40%

Measurement Scale of Rate of Agreement: 0–20%: radically different; 20–40%: numerous major scientific disagreements present; 40–60%: few major scientific disagreements present; 60–80%: only minor scientific disagreements present; 80–100%: absolute scientific agreement. In blank fields, no information is available

*APACHE*-*II score* Acute Physiology and Chronic Health Evaluation II score; *CECT* Contrast-enhanced computed tomography

**Fig. 2:**
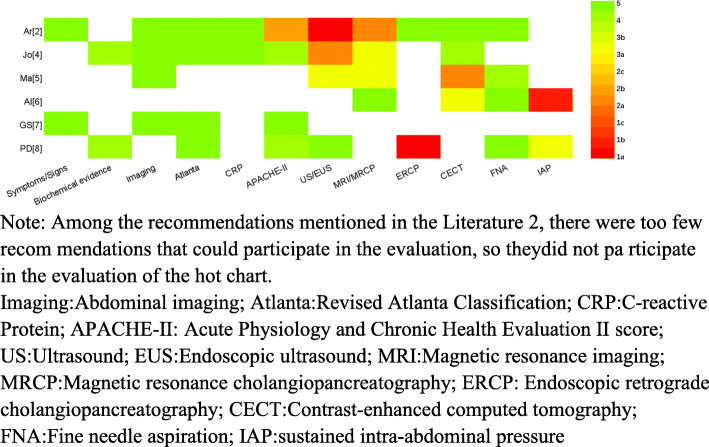
Distribution of the highest level of evidence to support similar recommendations for diagnosis of acute pancreatitis among included guidelines

Four guidelines [2, 3, 7, 8] recommend abdominal pain as a reference indicator for the diagnosis of severe pancreatitis; the other three only mention the symptom. The recommendations of the three guidelines [3, 7, 8] for abdominal pain (sustained attacks, severe and acute episodes of epigastric pain usually radiating to the back) had a high similarity (80–100%) to the reference recommendations. The cited levels of evidence were only grade 5 [2, 7]. Six guidelines [2–5, 7, 8] recommend serum lipase activity (or amylase) at least three times the upper normal limit as a reference indicator for the diagnosis of severe pancreatitis, among which three guidelines make an explicit recommendation [4, 5, 8], while the remaining three guidelines only casually recommend it [2, 3, 7]. Five guidelines [3–5, 7, 8] recommending serum lipase activity (or amylase) at least three times the upper normal limit had a high degree of similarity (80–100%) to the reference recommendation, and the reference level of evidence was up to grade 4 [4, 8]. In six guidelines [1–5, 7, 8], the characteristics of abdominal imaging are recommended as reference indicators for the diagnosis of severe pancreatitis. Three of these guidelines make explicit recommendations [4, 5, 8], and the remaining three guidelines merely mentioned these characteristics in the content [2, 3, 7]. The recommendations of five guidelines [3–5, 7, 8] for the characteristic findings from abdominal imaging had a high similarity (80–100%) to the reference recommendation. The reference level of evidence, cited in only four of the guidelines, was a low grade 5 [2, 4, 5, 7].

#### Severity classification of AP

Five guidelines [2–4, 7, 8] recommend the revised Atlanta classification as a reference indicator for the diagnosis of severe pancreatitis, of which two guidelines make explicit recommendations [2, 4] and the remaining three only mentions the classification in the content [3, 7, 8]. The recommendations of four guidelines [3, 4, 7, 8] for the revised Atlanta classification had a high similarity (80–100%) to the reference recommendation. The reference level of evidence cited in four of the guidelines was grade 5 [2, 4, 7, 8]. Four guidelines [2–4, 8] recommend CRP as a reference indicator for the diagnosis of severe pancreatitis, of which 3 guidelines make clear recommendations [2, 4, 8], and the remaining guideline only mentions CRP in the content [3]. The recommendations of two of the guidelines [4, 8] for CRP have a high similarity to the reference recommendation (80–100%). One guideline [3] demonstrated moderate similarity to the reference recommendation for CRP (40–60%). The reference level of evidence cited in one set of guidelines was grade 5 [2], and the reference level of evidence cited in another set of guidelines was a poor grade 5 [4]. Five guides [2–4, 7, 8] recommend the APACHE-II score as a reference indicator for the diagnosis of severe pancreatitis, three of which make explicit recommendations [2, 4, 7], and two of which only mention the score in the content [3, 8]. The recommendations of four of the guidelines [3, 4, 7, 8] for APACHE-II had a high similarity (80–100%) to the reference recommendation. One guideline had a reference level of evidence of only grade 5 [7], two guidelines had only grade 4 [4, 8], and one guideline had up to grade 2b [2].

#### Etiological diagnosis of acute pancreatitis

Seven guidelines [2–8] recommend US/EUS as the reference index for the diagnosis of the cause of severe pancreatitis, of which three guidelines make explicit recommendations [2, 4, 8], and 4 guidelines only mention US/EUS in the content [3, 5–7]. The recommendations of four guidelines [3–5, 8] US/EUS had high similarity (80–100%) to the reference recommendation, one guideline had moderate similarity [6] (40–60%), and one guideline had low similarity [7] (20–40%). The reference levels of evidence for one set of guidelines were grades 1a, 2a, 3a, and 5. Six guidelines [2–6, 8] recommend magnetic resonance imaging (MRI)/magnetic resonance cholangiopancreatography (MRCP) as a reference indicator for the diagnosis of severe pancreatitis, four of which make explicit recommendations [2–6, 8], while the remaining guideline only mentions MRI/MRCP in the content [3]. The recommendations for MRI/MRCP of these six guidelines had a high similarity (80–100%) to the reference recommendation. Four guidelines provide reference levels of evidence, the highest of which was grade 2a.

Five guidelines [2–5, 8] recommend endoscopic retrograde cholangiopancreatography (ERCP) as a reference indicator for the diagnosis of severe pancreatitis, of which three guidelines make explicit recommendations [4, 5, 8], while the remaining two guidelines only briefly mention ERCP in the content [2, 3]. The ERCP recommendations of four studies had high similarity to the reference recommendation (80–100%) [3–5, 8]. Only two guidelines cited reference levels of evidence for ERCP, one with a grade 5 level of evidence [2], and the other with grade 1a [8].

#### Diagnosis of AP comorbidities

CECT is recommended as a reference indicator for the diagnosis of severe pancreatitis in seven guidelines, four of which make explicit recommendations [4–6, 8], and 3 guidelines only mention CECT in the content [2, 3, 7]. The recommendation of CECT in five guidelines [3, 5–8] had high similarity to the reference recommendation (80–100%), and that of one guide [4] had a moderate similarity to the reference recommendation (40–60%). Only grade 5 evidence was cited in one guideline [2], grade 4 evidence is cited in one guideline [4], grade 2a in 1 guideline [5], and grade 3a in one guideline [6]. All seven guidelines recommend the collection of infections and fine needle aspiration (FNA) as a reference indicator for the diagnosis of severe pancreatitis after necrosis or infection, of which five guidelines make explicit recommendations [2, 4, 6–8], while the remaining two guidelines mention this procedure in the content [3, 5]. Four guides have a high similarity to the reference recommendations of FNA (80–100%). The level of evidence cited was grade 5 in one guideline [2], grade 4 in two guidelines [3, 5], and grade 5 in two guidelines [6, 8]. Four guidelines [3, 5, 6, 8] recommend continuous abdominal pressure (IAP) > 20 mmHg for the diagnosis of abdominal syndrome (ACS) as a reference indicator for the diagnosis of severe pancreatitis, two of which make explicit recommendations [3, 6], and the other two guidelines only mention this criterion [5, 8]. Although guideline [2] was the standard literature for similarity evaluation, it did not mention the diagnosis of abdominal cavity syndrome (ACS) with continuous intra-abdominal pressure (IAP) > 20 mmHg, so no similarity scores could be obtained for this recommendation. One guideline cited a level of evidence of grade 1b [6], and one guideline cited a level of evidence of 3a [8].

## Discussion

Compared with other disease-specific guidelines, the establishment of a diagnostic guideline for severe pancreatitis may be a more complex issue; these guidelines are a global concern managed by a variety of disciplines. This study found that in the initial diagnosis of acute pancreatitis, severity classification, aetiology diagnosis, and comorbidity diagnosis, there are irrational references to evidence, many indicators for the classification of acute pancreatitis, unclear standard descriptions, and poorly comprehensive recommendations for the determination of the aetiology of acute pancreatitis. The quality evaluation of the included guidelines with AGREE II demonstrated that the quality of the diagnostic guidelines was uneven. Even if there was a high degree of heterogeneity in different domains for the same guideline, the scores of only two sets of guidelines were high, indicating that they could be recommended for clinical use.

### Recommendations for the diagnosis of acute pancreatitis

Although most scholars do not believe that the diagnosis of acute pancreatitis should follow a certain procedure, clear procedures would help guide users and readers to better understand and study the diagnosis of acute pancreatitis [3]. One set of guidelines suggests [3] that the diagnosis of acute pancreatitis should include a preliminary diagnosis, severity classification, aetiology diagnosis, and comorbidity diagnosis. Therefore, in this study, we reviewed the recommendations of this guideline for the diagnosis of acute pancreatitis, and the content was combed.

#### Heterogeneity in the preliminary diagnosis of acute pancreatitis

Of the seven guidelines included, the similarity of the recommendations for abdominal pain, biochemical evidence, and imaging characteristics as criteria for the primary diagnosis of acute pancreatitis (Table 3) was high (80–100%) (Table 4). The main reason for the high similarity is that most guidelines [1, 2, 6, 7] recommend abdominal pain and chemical and imaging evidence as the standards for the primary diagnosis of AP, which were identical to the standards of the Atlanta classification [12]. However, there is obviously much room for improvement for the source of evidence and the standardization and rigor of the initial diagnosis. For example, although three guidelines recommend initial diagnostic criteria for acute pancreatitis, no source of evidence is provided [3, 7, 8], a clear indication of a lack of rigorousness. One of the guidelines focuses on the diagnosis of the comorbidity of severe pancreatitis [6]; however, there is a lack of recommendations for the preliminary diagnosis of acute pancreatitis. Three guidelines explicitly recommend biochemical evidence and imaging criteria for diagnosing acute pancreatitis [4, 5, 8] but lack recommendations for abdominal pain. The Japanese guidelines on biochemical examination suggest that two test strips for urinary pancreatinogen can be used for rapid diagnosis of acute pancreatitis in the future; however, the test strip is not commercialized in Japan, and therefore, it is currently not recommended for use [5].

#### Heterogeneity of acute pancreatitis severity classification

(1) There are many recommended grades for the severity of acute pancreatitis: of the seven guidelines included, a total of 8 grades are available for reference. The Atlanta classification, C-reactive protein level, and APACHE-II score are recommended by more than 3 guidelines, while other indicators are only recommended or mentioned in 1–3 guidelines. This is related to the lack of high-quality studies comparing the pros and cons of these acute pancreatitis severity indicators. (2) The clinical outcome indicators used in the guidelines to support the evidence of recommendation are significantly different. For example, in guideline [2], the supporting evidence for CRP is a diagnostic study [13], and the researchers use the Atlanta classification (2012) as a reference to determine the accuracy of CRP in the diagnosis of severe pancreatitis. Similarly, in another guideline [4], the original literature supporting the evidence for CRP [14] is a review in which the author refers to the use of the Atlanta classification (1992) in a meta-analysis of the accuracy of CRP in the diagnosis of severe pancreatitis. The Japanese guidelines [5] recommend the JPN severity score and BISAP as the criteria for the classification grade of acute pancreatitis, and the reference is a systematic review [15]. In this review, the authors evaluate the pros and cons of these two indicators with reference to persistent multiple organ failure. The evidence supporting the APACHE-II score in guideline [3] uses mortality as a reference evaluation. Obviously, guideline users and readers cannot distinguish the pros and cons of these indicators given the heterogeneity of evidence and references. (3) The description of a standard for acute pancreatitis grading indicators is not sufficiently clear. For example, one guideline suggests that we can refer to indicators such as the APACHE-II score, CT grading, modified CT severity index, CRP, and Ranson, but no specific reference values are given, and the corresponding evidence to support them [3] is also scarce. Four guidelines recommend C-reactive protein as an indicator of the severity of acute pancreatitis, two of which provide specific reference values for CRP [4, 8], two guidelines [2, 3] do not, and two guidelines do not provide evidence to support CRP [3, 8]. One guideline [7] refers to the revised Atlanta classification, the JPN severity score, urea and APACHE-II scores, but no specific evidence is given to support them. (4) The evidence cited is not sufficiently reasonable. For example, one guideline [4] supporting the recommendation of CRP provides evidence; however, the original reference is secondary literature, and the original literature [14] is a review in which the author refers to the Atlanta classification (1992), which was used for a meta-analysis of the accuracy of CRP in the diagnosis of severe pancreatitis. The original text did not provide the heterogeneity of the meta-analysis, and the stability of the results cannot be judged. The Japanese guidelines recommend the JPN severity score and BISAP as grades for the diagnosis of acute pancreatitis. The reference for this evidence is a systematic review [15], in which the authors used continuous multiple organ failure as a reference and found that the JPN severity score and BISAP had high specificity in the diagnosis of multiple organ failure within 48 hours of admission. However, the combined BISAP data in this review were significantly heterogeneous (*I*^2^ = 93.2), so the results are unstable.

Obviously, in the future, international organizations are expected to establish a global consensus on the severity classification of pancreatitis, so as to end the current confusion.

#### Heterogeneity in the aetiological diagnosis of acute pancreatitis

(1) The recommendations for the diagnosis of the aetiology are not comprehensive; it is important to identify the cause of acute pancreatitis for its early treatment. The main cause of acute pancreatitis is gallbladder or bile duct stones, followed by alcohol use and smoking, other rare drug use-related causes, and factors such as hypercalcemia, hypertriglyceridemia, surgery, and trauma [16, 17]. Therefore, the recommendations in the guidelines for the diagnosis of acute pancreatitis aetiology focus more on the examination of the biliary tract and gallbladder (Table 3), including US/EUS, MRI/MRCP, ERCP, and liver function tests. Only two recommendations are aimed at other rare factors, including hypertriglyceridemia and family genetic factors. The possible reason for this lack of focus may be that other rare factors that cause acute pancreatitis are relatively scarcely encountered in clinical studies, and the guideline developers did not systematically document the factors related to acute pancreatitis. (2) The evidence cited is unreasonable. For example, for the MRCP recommendations, one guideline [4] cites a high-quality meta-analysis [18], while two guidelines [3, 8] recommend MRCP to check the biliary system; however, no evidence was provided. One guideline [5] provided 3 pieces of evidence in support of MRCP, but one piece of evidence focused on endoscopic ultrasound and was not related to MRCP [18], likely a citation error. In two guidelines [4, 5], the ultrasound evidence is a retrospective case analysis, one guideline [2] uses a review, and three guidelines that recommend ultrasound or ultrasound endoscopy provide no evidence [3, 7, 8].

#### Heterogeneity in the diagnosis of acute pancreatitis comorbidities

For acute pancreatitis comorbidities such as pancreatic necrosis, peritoneal effusion, pseudocyst, and wall necrosis (WoN), the 7 included guidelines recommend the selection of CECT for a clearer diagnosis. If the effusion or necrotic tissue is considered to be infected, all guidelines recommend fine needle aspiration (FNA) to confirm the diagnosis of bacterial culture. Although the evidence of the recommendations for the diagnosis of acute pancreatitis comorbidities lacks high-quality studies, the evidence provided by the guidelines is very different. For example, for the CECT diagnosis of pancreatic necrosis, the evidence of three guidelines [4–6] is a retrospective case analysis, and the evidence of one guideline [2] is a review; three guidelines [3, 7, 8] recommending CECT do not provide evidence. The evidence of one guideline [2] recommending FNA is a prospective study, two guidelines [4, 5] use a retrospective observational study, one guideline [6] uses expert consensus, and one guideline [8] directly quotes another guideline. All of this evidence was published before 2014. The main reason for this evidence heterogeneity is that the guideline authors did not conduct a systematic search of evidence.

In summary, this study puts forward suggestions for improving the procedures in the diagnostic guidelines for acute pancreatitis: (1) It is recommended that in the future guidelines, the concept of diagnostic procedures should be introduced, which should be standardized to logically help guide users to diagnose acute pancreatitis more clearly; (2) greater attention should be paid to the issue of evidence citation in the diagnosis of acute pancreatitis, with a focus on a comprehensive and systematic retrieval of existing evidence and fair citations; (3) the graded indicators of acute pancreatitis should be clearly described so that readers and clinical practitioners can use the guidelines accurately; and (4) the recommendations for the diagnosis of acute pancreatitis generally lack high-quality research support, and it is recommended that research teams participating in the development of guidelines pay attention to and carry out corresponding high-quality research.

### Quality evaluation of guidelines for acute pancreatitis

We need to make improvements in the following areas. Stakeholder engagement reflects how well the guideline represents the views of its intended users, including patients. Implementation of the guideline requires the contribution and expertise of a multidisciplinary medical team, including patients. As guidelines develop, patients’ perspectives, expectations, and preferences for healthcare become increasingly important. However, no guidelines have yet provided detailed information on the involvement of patients or their representatives. The rigor of guideline development is the most critical area and will affect the confidence of the implementation of the guidelines [19]. Unsystematic development can easily lead to poor-quality guidelines [20]. Three guidelines [2, 3, 8] do not describe the literature search and selection methods, and it is not clear how to evaluate the evidence or develop the recommendations. The reason for the lower score may be the lack of methodological consultation [19], unfamiliarity with the guidelines and bad reporting [21], or poor performance of the external peer review and update process [22]. The low level of the Clarity and Expression domain reflects an inadequate reporting of key information in the full text, which suggests that attention should be paid to improving the quality of reporting. In 2016, the AGREE II group formulated a new checklist to improve the quality of reporting in guidelines [23], which should be used by the developers of acute pancreatitis guideline in the coming days.

The low applicability scores in the applied areas indicate that guideline developers have not paid enough attention to potential obstacles affecting the actual implementation of the recommendations [24]. Preliminary trials should be conducted to ensure feasibility before publication and find obstacles in the application. In order to facilitate the promotion of guidelines, the relevant supporting documents and suggestions should be provided in guidelines and the potential impact of recommendations on resource input should be discussed in guidelines.

The results of our research required the careful re-evaluation of existing guidelines and improvements in the quality of formulation and reporting in practice. First, in addition to being familiar with the guideline development standards, instrument developers should apply these tools and, wherever possible, describe them in the guidelines [25]. Second, the guidelines should be rigorously reviewed for eligibility for quality standards before publication and periodically re-evaluated after publication to make necessary updates. Journal editors should set higher standards for peer review, and only guidelines that meet quality standards should be considered for publication. Third, when the evidence supporting the recommendations is inadequate or incomplete, the development of guidelines can also be based on a reliable consensus statement, which is a robust and transparent process [26, 27]. Furthermore, attention should be paid to recommendation disputes between different countries. In addition, strengthening international cooperation among guideline developers will help minimize overlapping efforts and resolve disputes [24]. Finally, the guide team should address obstacles, provide advice on who, when, where, and how to provide as much specificity as the evidence allows.

In general, our research has some advantages. First, our authors come from different backgrounds, including clinical experts and methodologists with rich experience in the evaluation of clinical guidelines, which improves the reliability of our findings. Second, the different areas have been appropriately weighed to arrive at an overall assessment and recommendations. Nonetheless, our research has several limitations. The exclusion of guidelines published in languages other than English and Chinese or in other formats (i.e., books, brochures, or government documents) may lead to an underrepresentation of guidelines from less developed countries. Second, the AGREE II tool focuses on guideline development methods and reporting transparency but cannot assess the potential impact of recommendations on patient outcomes [28, 29]. In addition, poor performance of guidelines for severe pancreatitis may be related to insufficient reporting quality, which may underestimate the score we evaluated.

## Conclusion

The diagnostic procedures in all guidelines for acute pancreatitis have unreasonable references to evidence, there are many grades of acute pancreatitis, the standard description is not sufficiently clear, and the recommendations for the diagnosis of the aetiology are not sufficiently comprehensive. The quality of the guidelines for the diagnosis of acute pancreatitis is uneven, and there is much room for improvement, especially in the areas of applicability, stakeholder involvement, and editorial independence. Acute pancreatitis guidelines should develop recommendations for high-quality evidence while minimizing external error with convincing methodological rigor and transparency.

## Supplementary Information

**Additional file 1: **Table S1**.** Levels of evidence based on the Oxford Centre for Evidence-Based Medicine

## Data Availability

All authors agree to share the data of this review, which can be obtained by contacting the corresponding authors. Email: sundali2018@126.com.

## Abbreviations

GRADE: Grading of Recommendations Assessment Development and Evaluation
OCEBM: Oxford Centre for Evidence-based Medicine-Levels of Evidence
U.S.: The United States
UG: Ungraded
NG: Not given
ICC: Intra-class correlation coefficient
AGREE II: The Appraisal of Guidelines for Research and Evaluation II
MSRA: Measurement Scale of Rate of Agreement
WSES: World Society of Emergency Surgery
AISP: The Italian Association for the Study of the Pancreas
JSHBPS: Japanese Society of Hepato-Biliary-Pancreatic Surgery
SCGS: CAIM Specialized Committee of General Surgery, Chinese Association of Integrative Medicine
PDGCSG: Pancreatic Disease Group of Chinese Society of Gastroenterology
EBCJP: Editorial Board of Chinese Journal of Pancreatology
EBCJG: Editorial Board of Chinese Journal of Gastroenterology DDC Digestive Disease Committee
CAIM: Chinese Association of Integrative Medicine
NSTSP: National Science and Technology Support Program
