# Supplementary material for: Appraisal of the diagnostic procedures of acute pancreatitis in the guidelines
Source: Syst Rev. 2021 Jan 9;10:17. doi: 10.1186/s13643-020-01559-4 (PMC7796583; doi:10.1186/s13643-020-01559-4)
Supplement: Supplementary file 1 — Additional file 1: Table S1. Levels of evidence based on the Oxford Centre for Evidence-Based Medicine [file 13643_2020_1559_MOESM1_ESM.docx]

Table S1 Levels of evidence and grades of the recommendations based on the Oxford Centre for Evidence-Based Medicine

| Recommendations | Grade | Description |
| --- | --- | --- |
| A | 1a | SRs based on RCTs (with homogeneity) |
|  | 1b | Single RCT study (with narrow confidence interval) |
|  | 1c | "All or no" evidence (by death of all patients before treatment, and survival of patients after treatment. Or some patients died before treatment, and no patients died after treatment) |
| B | 2a | SRs based on cohort studies (with homogeneity) |
|  | 2b | Single cohort studies (including low-quality RCT studies, e.g. < 80% follow-up) |
|  | 3a | SRs based on case-control studies (with homogeneity) |
|  | 3b | Single case control study |
| C | 4 | Case Reports (Low Quality Cohort Studies) |
| D | 5 | Expert opinion or comment |
| UG: Unclassified. |  |  |
